# Medication-Related Osteonecrosis of the Jaws: Two Center Retrospective Cohort Studies

**DOI:** 10.1155/2019/8345309

**Published:** 2019-03-18

**Authors:** Milan Petrovic, Drago B. Jelovac, Svetlana Antic, Marija Antunovic, Nikola Lukic, Melvil Sabani, Joerg Mudrak, Zoran Jezdic, Ana Pucar, Aleksandar Stefanovic, Cedomir Kuzmanovic, Danilo Nikolic, Vitomir Konstantinovic

**Affiliations:** ^1^Clinic for Maxillofacial Surgery, School of Dental Medicine, University of Belgrade, Serbia; ^2^Center for Radiological Diagnostics, School of Dental Medicine, University of Belgrade, Serbia; ^3^Laboratory for Anthropology, School of Medicine, University of Belgrade, Serbia; ^4^Faculty of Medicine, Clinical Centre of Montenegro, Podgorica, Montenegro; ^5^Faculty of Medicine, University of Belgrade, Serbia; ^6^Joerg Mudrak, Private Clinic, Rotenburg an der Fulda, Germany; ^7^Clinic for Periodontology and Oral Medicine, School of Dental Medicine, University of Belgrade, Serbia

## Abstract

This retrospective cohort study aims to describe characteristics of patients with MRONJ, to identify factors associated with MRONJ development, and to examine variables associated with favourable outcome. Totally 32 patients were followed and observed: 21 females and 11 males, in the age range 35-84 in the period from 2009 to 2018. Clinical, radiological examination (Orthopantomograph and CBCT) and biopsy were performed in order to achieve diagnosis. Demographic and clinical variables were taken into consideration: sex, age, primary disease, medication type, mode of delivery, anatomic location, drug treatment duration, timing of tooth extraction, chemotherapy, presence of bone metastasis, aetiology of MRONJ, disease stage, and treatment modality. MRONJ developed under osteoporosis and malignant disease in 11 and 21 patients, respectively. MRONJ development was triggered by tooth extraction or trauma in 30 out of 32 cases, whereas the two patients developed MRONJ spontaneously. Stages I, II, and III were confirmed in 5 (16%), 18 (58%), and 9 (28%) patients, respectively. Mandible was affected in 23 (72%) patients. MRONJ was treated in our department by conservative and surgical modality. In this study we found that 65% of all patients were classified in the cured/improvement group and 35% in the stable/progression group. The female gender, osteoporosis as primary disease, oral regime intake, shorter period on BPs, earlier stage of disease, and specific anatomic localisation (frontal and premolar maxilla) were factors associated with better response to therapy and favourable clinical outcome. Comprehensive treatment protocol and further randomized studies are necessary for further improvements.

## 1. Introduction

Bisphosphonates (BPs) are medications, frequently prescribed in therapy of various pathological conditions affecting bones. On the other hand, they might give side effects. Furthermore, bisphosphonate related osteonecrosis of the jaws (BRONJ) has become very popular topic among clinicians because of its unreachable nature and unpredictable outcome. It was presented in the literature for the first time in 2003 as one of the most serious side effects of BPs therapy [1]. Later on, the other drugs have been related to this serious disease. Medications that cause this severe, difficult to treat condition of the jaws are now grouped into two categories: BPs and non-BPs (considering other antiresorptive or antiangiogenic medications) [2]. There are several similar names describing this pathological condition apart of BRONJ: MRONJ (Medication Related Osteonecrosis of the Jaw), ARAONJ (Anti-Bone Resorptive Agents Osteonecrosis of the Jaw), etc. [3]. Despite being in the focus of the research, its exact aetiology, pathogenesis, and adequate treatment protocol remain controversial. [2] Regardless of great scientific understanding and plenty of published proposals no consensus regarding the treatment has been achieved yet.

MRONJ has been the most discussed adverse effect of bisphosphonate therapy in the last decade. This phenomenon was for the first time detected and described by Marx [1] as a painful and nonhealing bone exposure.

According to the criteria defined by American Association of Oral and Maxillofacial Surgeons (AAOMS), MRONJ presents with exposed necrotic bone in maxillofacial region for more than 8 weeks in patients without history of radiation therapy to the orofacial region and without obvious metastatic lesion in the jaw, which are currently on or were taking antiresorptive or antiangiogenic agents [2]. AAOMS also defined staging system for MRONJ and proposed treatment protocol for each stage [2].

It is noticed that MRONJ appears corporately with infection and usually is induced by local trauma, such as tooth extraction or other invasive dental procedures. There are also clinical evidences that the risk of developing MRONJ increases proportionally to the dosage and duration of medication intake and it is greater in patients with oncological disease, particularly in combination with chemotherapy [2].

To our knowledge, the cases of MRONJ in the population of Serbia and Montenegro have not been reported and described in detail so far.

The purpose of this study is to present MRONJ patients from Serbia and Montenegro taking into consideration demographic and clinical findings as well as treatment outcome.

In order to accomplish this aim the following tasks are set: (1) to describe the characteristics of a cohort of patients with MRONJ, (2) to identify the factors associated with the development of MRONJ, and (3) to identify the variables associated with favourable outcomes.

## 2. Material and Methods

### 2.1. Study Design and Sample

To emphasize the purpose of the research, the researchers designed and implemented a retrospective longitudinal cohort study and included patients diagnosed with MRONJ. Patients who did not showed for the follow-up for more than 6 months after treatment were excluded from the study. Total of 32 patients with oral side effects after bisphosphonates and Sutent (Sunitinib) use with MRONJ were diagnosed and treated in 9 years period (2009 and 2018) in two University Hospitals (Clinic for Maxillofacial Surgery, School of Dental Medicine in Belgrade (Serbia) and Podgorica (Montenegro)). Twenty-one were women. The mean age at presentation was 59 years (SD ±11.8 yrs). The age of these patients (pts) ranged from 37 up to 84, and male to female ratio was shifted more towards females (21:11). Patients were referred to our departments because of nonhealing wound in one of the jaw, after tooth extraction. None of these patients had a history of radiation therapy to the orofacial region. Twenty-one of them had underlying malignant disease. Underlying oncological history included eleven patients with breast carcinoma, four patients with prostate carcinoma, four multiple myeloma patients, and one patient with medullar thyroid carcinoma (MTC) and renal cancer. Fourteen pts out of 21 pts with malignant disease have got bone metastasis before onset of MRONJ. Seventeen out of 21 pts with malignant disease were on chemotherapy. The most commonly used drug was Zoledronate (Table 1).

**Figure 1:**
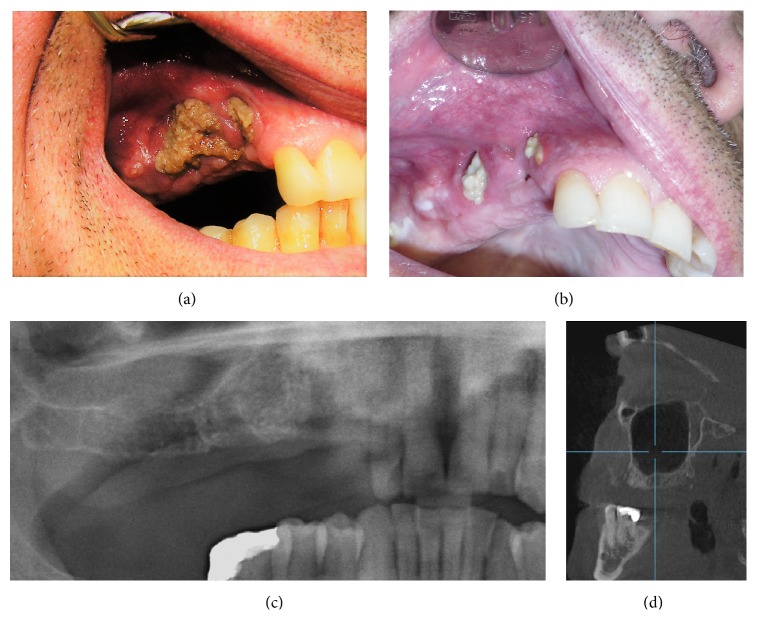
(a) Patient with medullary thyroid cancer, before and after conservative treatment. Patient just performed brushing exposed bone with stick or spatula with chlorhexidine 0,12% solution. Significant improvement (b). Panoramic radiography (c) and cone beam computer tomography rolled out bone sequestrum. (d) Scanora 3Dx device (*On Demand Software Cybermed, Seoul, Korea.)*

**Figure 2:**
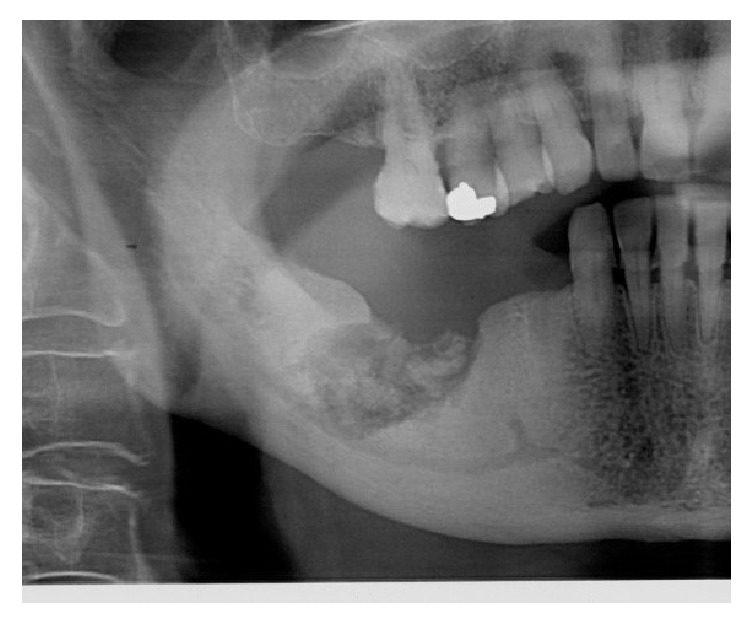
Stage III, MRONJ in the right mandibular corpus. Radiolucency with sequestrum.

**Figure 3:**
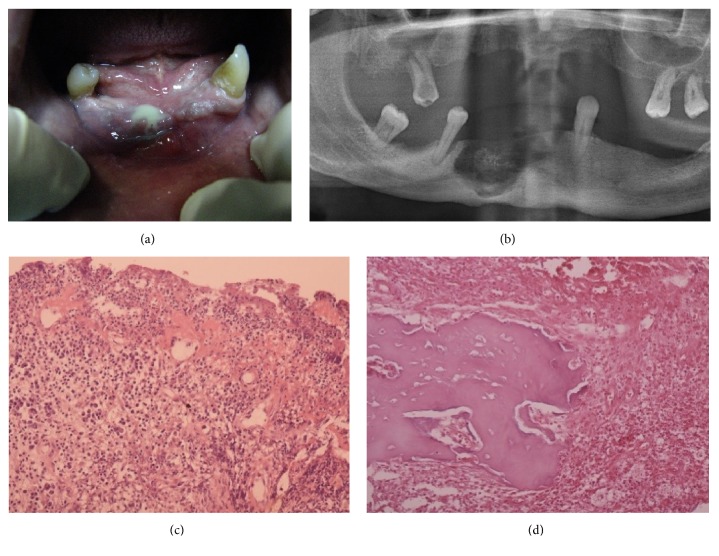
Stage III, MRONJ, with “iceberg” phenomena. (a) Small area with pus in the frontal region of the mandible. (b) OPG with significant bone destruction and sequestrum in frontal region. (c) Granulation, connective tissue with small abscess infiltrated with neutrophils, plasma cells, and lymphocytes. The necrotic masses at surface. (d) Necrotic tissue with connective tissue, neutrophils, plasma cells, and granulocytes (HE, x200)

**Figure 4:**
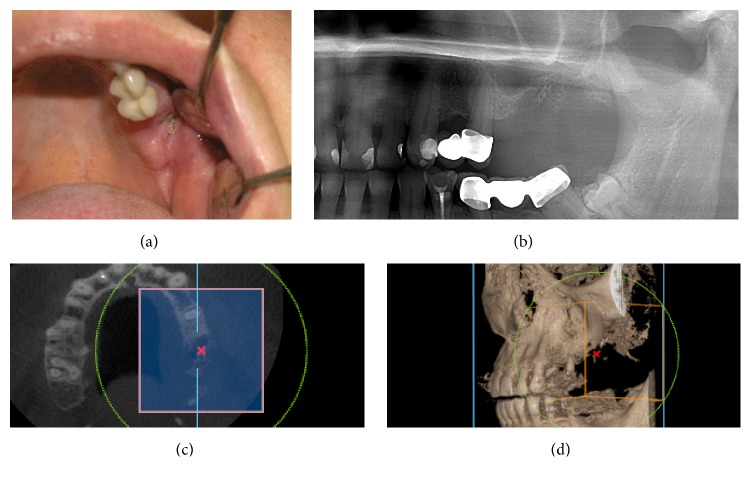
Stage I MRONJ. (a) Intraoral detail. (b) Orthopantomograph and CBCT 3D mode and cross section analysis excluded any bony sequestrum. (c) Axial view of CBCT confirmed our findings. (d) 3D mode view in Scanora 3D software is especially useful tool. Using 3D zoom tool, it is very easy to eliminate other structures which could interfere with region of interest.

**Figure 5:**
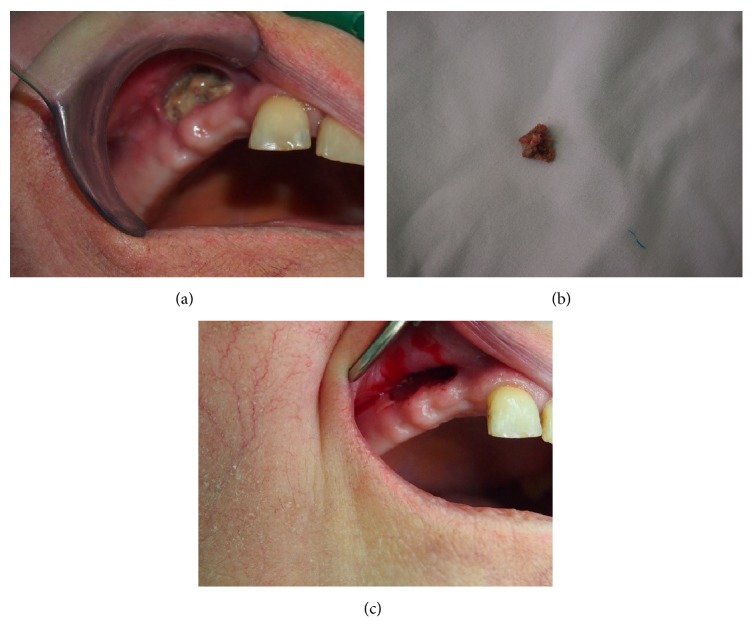
MRONJ stage III. (a) Clinical appearance of bone sequestrum in the maxillary area. (b) Sequestrum removed. (c) Favourable wound healing.

**Figure 6:**
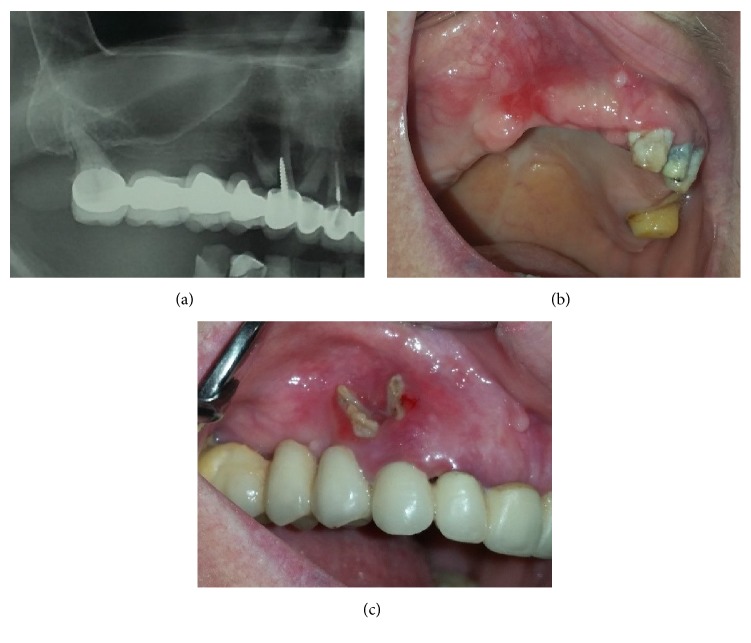
(a) Radiological appearance of MRONJ. (b) Lesion cured. (c) Clinical appearance.

The stage system was according the AAOMS criteria [2]. After clinical diagnosis, patients undergo orthopantomography and targeted CBCT. Radiographic findings showed either persisting and nonhealing tooth socket after extraction, often accompanied with thickening of lamina dura, or radiolucent osteolytic zones and superficial bone defects. To define preoperative stage and to achieve the most predictable treatment outcome, targeted CBCT scan was performed (SCANORA 3Dx®, Tuusula, Finland). The analysis of the bone structure was performed by using ROI (region of interest) and profile function to confirm/exclude bone sequestration. Dento-maxillofacial radiologist evaluated all images (Figures 1–6).

Conservative treatment modality was utilized for stage 0-2 patients which included swab, systemic antibiotic treatment, systemic administration of tocopherol and pentoxifylline prescription, and brushing the lesion with spatula or spatula (stick) for swab sampling in conjunction with solution of chlorhexidine 0,12 % (oral antiseptic mouth rinses are recommended). Surgical approach was performed for stages 2 and 3.

Patients without sequestration, either clinically or radiologically, were observed and just medications were prescribed (Pentoxifylline tablets 400 mg per or twice daily). Antibiotic treatment was given according to swab findings. Mouth rinses (chlorhexidine solution 0,12%) were recommended in periodical regime twice a day. For stage 3 disease patients, sequestrectomy or radical bone removal was performed. They were not suitable candidates for major reconstructive microsurgical reconstruction due to significant medical comorbidities.

This study met the criteria for exemption by the institutional review board.

## 3. Study Variables

### 3.1. Predictor Variables

The predictor group of variables involved a varied group of variables divided into the following groups: (1) demographic, (2) clinical data, and (3) treatment modality.

The demographic variables involved sex and age at the time of MRONJ diagnosis, the underlying disease (malignant or nonmalignant), drugs used (BPS or antiangiogenic agent), duration of exposure and way of administration, chemotherapy, presence of bone metastasis, and trigger for MRONJ developing (tooth extraction or trauma). Patients were divided into the following groups regarding the way of BPs administration: (1) only intravenously (IV), (2) IV+ oral (PO), and (3) PO. All patients were clinically evaluated by a maxillofacial surgeon.

Clinical data considered anatomic location of disease (mandible, maxilla, or both) and disease stage at the first presentation.

According to the treatment modalities patients are divided into the following groups regarding the type of treatment they had: conservative and surgical.

### 3.2. Outcome Variable

The clinical outcome was divided in two groups: cured/improvement (resolution or positive change of stadium 6 months after treatment) and stable/progression (stability or negative change of stadium 6 months after treatment).

The patients were considered cured if complete healing of mucosa over the exposed bone with pain relief had occurred.

The patients were considered improved if they were better regarding the symptoms or had progressed to a lower disease stage after the treatment [4–6]. If their disease had not advanced to a higher stage, patients are considered stable.

Progression was considered if patients experienced more severe pain, inflammation, or more bone exposure after the treatment, with advances to a higher stage [4–6].

### 3.3. Statistical Analysis

A Microsoft Excel database was used for data collection and storing. The descriptive and analytic statistics were computed. For each variable of interest, logistic regression analysis was used to examine the association between the outcome and the factor. All variables were then involved in multivariable logistic regression analysis to examine joint effects of those factors on the outcome. Statistical significance was set at P <0.05 SPSS version 18.0 software was used for statistical calculations.

## 4. Results

Twenty-six patients totally were on IV regime (81.25%) (Table 1). Only six pts (18.8 %) took bisphosphonates orally. Five of them were on IV+PO regime (15.6%). Only one patient received both zoledronic acid and sunitinib concomitantly.

In twenty-three cases (72%) lesion occurred in the mandible (6 in the anterior sextant and 18 in the posterior), in 8 cases (25%) in the maxilla (1 in the anterior sextant and 7 in the posterior), and in 1 (3%) in both jaws (all occurring in the posterior sextant). The average duration of medical agents exposure varied between the different type of drugs. (Table 1).

Twenty-three patients (71.8%) developed lesions while actively receiving bisphosphonate therapy.

The time range for the patients who developed disease after terminating BPs treatment was between 4 and 84 months.

The main event leading to MRONJ development was tooth extraction in 29 cases (91%), implant extraction in one case (3.1%), and spontaneous type occurred in two cases (6%) (Table 1).

21 bone biopsies were examined by pathologist. The findings were consistent with a diagnosis of chronic osteomyelitis that ruled out malignancy. Necrotic spaces showed empty osteocyte lacunae with medullary spaces and surrounding tissue colonized by Actinomyces like microorganisms. These germs were confirmed in 10 cases (31%); however no statistical significance with clinical outcome and other parameters were found (data not shown in table).

Eighteen patients (56.2 %) underwent surgery after initial antibiotic treatment, whereas fourteen patients (43.8%) received only conservative treatment. Follow-up period range was 6 to 63 months (mean 32.4 ± 16.8).

The variables which are compared to clinical outcome are shown in Table 2. There was no statistical significance in examined variables (sex, age, underlying disease, stage, BPs intake groups, BPs treatment duration, aetiology, timing of tooth extraction regarding the cessation of medications related to MRONJ, and surgical approach between patients who experienced clinical improvement or healing and those who did not) (Table 2.)

Differences (but not statistically significant) are noted in the gender, underlying disease, stage, BPs consumption, BPs intake (way of administration), and surgical approach and timing of extraction.

The percentage of response to therapy for male patients was lower (54.5%) in comparison with female whereas treatment response was better (71.4%). No statistical significance was found in the treatment outcome between mandible and maxilla. However premolar and frontal region of the upper jaw responded excellently to the treatment (5 patients (100 % cured or improved) in comparison with symphyseal part of mandible (50%) and molar region of upper jaw (66%)).

No significant relationship was found between the stage of the MRONJ and the modality or the duration of BP therapy. However, patients who were on BPs therapy less than 12 months showed good response to treatment (5 patients, 80% cured or improved) (Table 1.) However, patients who were on BPs more than 12 months had more serious stage of MRONJ. Patient who was on BPs and Sutent concomitantly got the most severe stage of MRONJ of both jaws and was nonresponsive to the therapy. He developed oronasal and oroantral fistula.

The difference was observed between the group of pts who received BPs PO and only IV, but without statistical significance (Table 2).

Patients treated conservatively showed better response to therapy (11 patients, 78.6%), but not without statistical significance (Table 2).

The patients were staged as 0, I, II, and III, respectively, 0, 5, 18, and 9, regarding the staging system of AAOMS (Table 1) [7].

Disease outcome was considered clinically as cured/improvement and stable/progression 66% and 34%, respectively.

## 5. Discussion

MRONJ is a disease with very unpredictable outcome. There is a serious adverse effect of bisphosphonate therapy due to its incompletely clarified etiopathogenesis [2]. None of the proposed theories concerning etiopathogenesis of MRONJ has found scientific approval and confirmation [8]. These theories mostly explain MRONJ because of inhibition [5] of bone remodelling process, antiangiogenic potential of bisphosphonates, direct toxic effect on oral epithelium, or their associated negative effects with infection and inflammation.

To consider MRONJ disease 2 parameters need to be evaluated: therapeutic indications and types of medication (Tables 1 and 2). Patients at risk of MRONJ are often subdivided into two subcategories: low risk (osteoporosis; oral medication) and high risk (cancer patients, etc.).

MRONJ was confirmed mostly in females (65.6%) which is previously reported [9]. One of the reasons which could explain the frequency of disease in females could be the higher incidence of breast cancer in females and skeletal metastasis. This disease presents one of the main indications for BPs therapy. The age and gender as risk factors are inconsistently presented in the scientific literature [10, 11].

Previous studies have reported that risk of developing MRONJ is higher in cases with intravenous administration of bisphosphonates, especially when more potent amino bisphosphonates are used, and increases with bisphosphonate therapy duration and dosage [2, 3].

The majority of patients in this study were on intravenous drug regime (81.2%). Patients from the study who were on BPs more than 12 months had more serious stage of MRONJ with lower percentage of favourable outcome. The risk is also higher in patients with underlying malignant disease [2, 12, 13], which was recorded for the majority of our patients (67%).

Most of the patients were on drugs related to MRONJ at the time of tooth extraction. The reason should be the lack of preventive measures and inadequate healthcare awareness of physicians regarding the side effect of MRONJ related drugs. Tooth extraction was the most frequent local risk, i.e., triggering factor for developing MRONJ along with other invasive dental interventions, which was also the case in this study group of patients (91%). Inadequate dentures that compress oral mucosa against bony prominences might also be the initiating factor for MRONJ development [14]. We got two patients with spontaneous MRONJ and one of them was cured. There is a lack of published cases with regard to the outcome of spontaneously developed MRONJ [15, 16]. There is no statistically significant association between the outcome and subsite of MRONJ in the jaws or primary disease on the other side. However, in our study premolar and frontal region of the upper jaw responded excellently to the treatment (100% cured or improved).

Besides patients with diagnosis of osteoporosis, all others from our study group had underlying malignant disease and were receiving high potent-nitrogen containing BPs or antiangiogenic medication in addition to chemotherapy, all of which, according to references data, increase risk of developing MRONJ. Some of the patients received corticosteroids, which also increased the risk of developing MRONJ and even more complicated its improvement [5, 10, 11].

Clinical findings in patients of current study are mostly in agreement with clinical findings in other reported researches, according to the illness staging. Although there were four patients in stage III with extensive osteolytic process, only one of them developed oroantral communication. That was the patient with advanced renal cancer who was on BPs and antiangiogenic drugs simultaneously. This simultaneous therapy is associated with less favourable outcome [10, 17]. The patients who present such a serious stage of MRONJ after concomitant BPs and chemotherapy could be eligible candidates for radical resection of the lesion and reconstruction with microvascular free flaps [18]. It should be highlighted that patients with MRONJ require meticulous preoperative assessment to avoid failure in treatment. MRONJ is located predominantly in mandible, which is logical, because maxilla is relatively spared due to better vascularisation and because of the difference in the anatomy [4, 19].

The panoramic radiograph is widely used imaging method in MRONJ patients, but panoramic image findings are not specific and could vary from predominantly sclerotic to predominantly lytic bone changes or their mixture [20, 21]. MDCT or MRI are considered the techniques with higher detectability and more sensitive methods for evaluation of bone necrotic process extension [22].

Preoperative assessment with cone beam computer tomography (CBCT) could be very powerful diagnostic tool in differential diagnosis, staging, prediction, and treatment planning [23].

However, in some cases due to similarity in the radiographic pattern, in some cases, it could be difficult to differentiate MRONJ from malignant process invasion, osteomyelitis, or consequences of radiotherapy to the orofacial region. Therefore, the history of radiation therapy and malignant process must be excluded before diagnosis of MRONJ is made. CBCT in this study was performed in majority of cases and showed high specificity to exclude bone sequestrum.

Generally, there are no sensitive characteristic histopathological features which would help MRONJ recognition [7]. Histological examination in all patients revealed osteonecrosis without pathological signs of malignancy. MRONJ was presented by avascular type of osteonecrosis. It was characterised with osteocyte-depleted bone lacunae. This specific type of avascular necrosis might have wide range of diversity [7]. However, the histology presents just piece of the complex puzzle in establishing diagnosis of MRONJ. Certainly, in this diagnostic step, it is very important to exclude primary malignant process or metastasis. On the other hand, sometimes it is difficult to assess whether biopsy should be taken, because unnecessary intervention may interrupt healing and exacerbate osteonecrosis. We believe that, after detailed anamnesis and clinical and radiographic examination, biopsy should be taken into consideration occasionally, in order to exclude potential malignant process which requires different kind of treatment, except in stages 0 and I.

Treatment protocol for MRONJ according to AOOMS recommendations is satisfactory, well accepted, and administered in our departments as well. Nevertheless, MRONJ is a condition without predictable and confident treatment outcome. It is well known that, in many cases, despite applied therapeutic measures, exposed necrotic bone cannot heal, especially when bisphosphonate therapy is not terminated. Discontinuation of bisphosphonate therapy cannot always be done, especially in oncologic patients, because that might interrupt malignant disease treatment. That certainly demands cooperation of oral or maxillofacial surgeon and oncologist. Otto and others have proposed the newest algorithm recently [12]. Clinical outcome in the literature varies. Some studies show high rate of success in treatment [24, 25]. According to one of the last reviews, good results were achieved in early stages, but heterogenous results in the advanced stages (50-100%) [26, 27].

Patients on bisphosphonate, antiresorptive, or antiangiogenic therapy must be educated how to recognise MRONJ or its symptoms, in order to contact their physician in time, so that they could be promptly referred to the oral or maxillofacial surgeon.

## 6. Conclusion

This paper presents and describes the ubiquitous inexplicable adverse effect of bisphosphonate therapy in patients included in this study. Total success rate of the management was satisfactory. Although no statistical significance was reached between patient related factors and final treatment outcome, the highest success rate is achieved if treatment starts in stage 1.

Medical doctors should be aware of this medical related disease and the role of dental evaluation before induction of these drugs as the cure rate is still not predictable. It must be highlighted that the collaboration between a physician and a dentist as well as a maxillofacial surgeon should be initiated before induction of these drugs. The future case control or randomized studies should be performed as there is the lack of these studies so far.

## Figures and Tables

**Table 1:** Demographic and clinical characteristics.

Demographic and clinical characteristics	
Characteristic	Value
Sample size	32 (100)
Gender	
Male	11 (34.3)
Female	21 (65.6)
Age	
Indication for medication	
Malignant disease	21 (65.6)
Nonmalignant disease	11 (34.4)
Medication risk factors	
Pamidronate	2 (6.2)
Zoledronic	24 (75)
Antiangiogenic agents	1 (3.1%)
Others (oral BPs)	6 (18.8)
Mode of delivery	
IV	21(65.6)
IV + Oral	5 (15.6)
IV + (IV + Oral)	26 (81.25)
Oral	6 (18.8)
Anatomic Location	
Mandibular	23 (71.9)
Maxillary	9 (28.1)
Maxilla and mandible	1 (3.1)
Treatment duration	
Zoledronate	33.87 ± 23.05
Pamidronate	6±0
Others (oral BPs)	34.9±20.87
Antiangiogenic agents	18± 0
Etiology of MRONJ	
Tooth extraction	29 (90.6)
Implant extraction	1 (3.1)
Spontaneously	2 (6.25)
Treatment modality	
Surgical	18 (56.2)
Conservative	14(43.8)

(i) Data presented as n (%) or mean ± standard deviation.

BPs, bisphosphonate; IV, intravenous subcutaneous; MRONJ, medication-related osteonecrosis of the jaw; PO, oral.

(ii) Treatment duration: exemption as values present number of months.

(iii) Sums to >100% because some patients used multiple medications.

(iv) IV: intravenous regime; IV+PO: intravenous + oral; some of the patients received drugs by both ways.

**Table 2:** Crosstab between study variables and clinical outcome.

*Study Variables*	*Clinical outcome*		total
	cured/improvement, N (%)	stable/progression N (%)	
*Sex*			
Male	6 (54.5%)	5 (45.5%)	11
Female	15 (71.4%)	6 (28.6%)	21
Total	21 (65.6%)	11 (34.4%)	32
*Age*			
<50 years	3 (60%)	2 (40%)	5
> 50 years	18 (66.7%)	9 (33.3%)	27
*Underlying disease*			
Osteoporosis	8 (72.7%)	3 (27.3%)	11
Malignant	13 (61.9%)	8 (38.1%)	21
*Stage*			
I	4 (80%)	1 (20%)	5
II	12 (66.7%)	6 (33.3)	18
III	5 (55.6%)	4 (44.4%)	9
*BPs consumption*			
<12 months	4 (80%)	1 (20%)	5
12 to 36	11 (61.1%)	7 (38.9%)	18
>36 months	6 (66.7%)	3 (33.3%)	9
*BPs intake*			
IV+(IV+PO)	16 (61.5%)	10 (38.7%)	26
PO	5 (83.3%)	1 (16.7%)	6
(IV+PO) +PO	9 (81.8%)	2 (18.2)	11
IV	12 (57.1%)	9 (42.9%)	21
*TIMING OF TOOTH EXTRACTION*			
During BPs therapy	16 (69.6%)	7 (30.4%)	23
After cessation of BPs therapy	5 (55.6%)	4 (44.4%)	9
*Chemotherapy*			
Yes	11 (64.7%)	6 (35.3%)	7
No	2(50%)	2(50%)	4
*Bone metastasis*			
Yes	9 (64.3%)	5 (35.7%)	14
No	4 (57.1%)	3 (42.9%)	7
*Localisation*			
mandible	15 (65.2%)	8 (34.8%)	23
maxilla	6 (66.7%)	3 (33.3%)	9
*Etiology*			
dental extraction-trauma	20 (66.6%)	10 (33.4%)	30
No dental extraction	1 (50 %))	1 (50%)	2
*Surgical approach*			
Surgical	10 (55.6%)	8 (44.4%)	18
Conservative	11 (78.6%)	3 (21.4%)	14

(i) Data presented as n (%) or mean ± standard deviation.

## Data Availability

The data used to support the findings of this study are available from the corresponding author upon request.
